# Exercise‐induced mitochondrial biogenesis coincides with the expression of mitochondrial translation factors in murine skeletal muscle

**DOI:** 10.14814/phy2.13893

**Published:** 2018-10-22

**Authors:** Takumi Yokokawa, Kohei Kido, Tadashi Suga, Tadao Isaka, Tatsuya Hayashi, Satoshi Fujita

**Affiliations:** ^1^ Laboratory of Sports and Exercise Medicine Graduate School of Human and Environmental Studies Kyoto University Kyoto Japan; ^2^ Faculty of Sport and Health Science Ritsumeikan University Kusatsu Shiga Japan

**Keywords:** Mitochondrial translation factor, mitochondrial biogenesis, exercise, skeletal muscle

## Abstract

The process of mitochondrial translation, in which mitochondrial (mt)DNA‐encoded genes are translated into proteins, is crucial for mitochondrial function and biogenesis. In each phase, a series of mitochondrial translation factors is required for the synthesis of mtDNA‐encoded mitochondrial proteins. Two mitochondrial initiation factors (mtIF2 and mtIF3), three mitochondrial elongation factors (mtEFTu, mtEFTs, and mtEFG1), one mitochondrial release factor (mtRF1L), and two mitochondrial recycling factors (mtRRF1 and mtRRF2) are mitochondrial translation factors that coordinate each translational phase. Exercise increases both nuclear DNA‐ and mtDNA‐encoded mitochondrial proteins, resulting in mitochondrial biogenesis in skeletal muscles. Therefore, mitochondrial translation factors are likely regulated by exercise; however, it is unclear whether exercise affects mitochondrial translation factors in the skeletal muscles. We investigated whether exercise training comprehensively increases this series of mitochondrial translation factors, as well as mtDNA‐encoded proteins, in the skeletal muscle. Mice were randomly assigned to either the sedentary or exercise group and housed in standard cages with or without a running wheel for 1 and 8 weeks. The expression levels of mitochondrial translation factors in the plantaris and soleus muscles were then measured. Exercise training concomitantly upregulated mitochondrial translation factors and mitochondrial proteins in the plantaris muscle. However, in the soleus muscle, these comprehensive upregulations were not detected. These results indicate that exercise‐induced mitochondrial biogenesis coincides with the upregulation of mitochondrial translation factors.

## Introduction

Mitochondrial function in the skeletal muscle is closely related to aging, sarcopenia, insulin resistance, and the progression of type 2 diabetes (Marzetti et al. 2010; Baker and Haynes 2011; Szendroedi et al. 2011). Accordingly, mitochondrial dysfunction is observed in the skeletal muscle of patients with type 2 diabetes (Kelley et al. 2002). In elderly people, muscular mitochondrial function and biomass are lower than in young people, and the rate of mitochondrial ATP production is positively correlated with whole body glucose tolerance (Short et al. 2005). Interestingly, muscle‐specific overexpression of peroxisome proliferator‐activated receptor gamma coactivator 1‐alpha, a master regulator of mitochondrial biogenesis, suppresses the decrease in exercise capacity and prevents insulin resistance during aging (Wenz et al. 2009). Therefore, interventions that improve mitochondrial function and enhance mitochondrial biogenesis are clinically significant in slowing aging, and preventing sarcopenia and insulin resistance.

Mitochondrial genes are encoded by both nuclear DNA (nDNA) and mitochondrial DNA (mtDNA). The mtDNA consists of approximately 16,000 base pairs encoding two ribosomal RNAs, 22 transfer RNAs, and 13 proteins (Anderson et al. 1981; Hällberg and Larsson 2014). These proteins form part of the mitochondrial respiratory chain complexes and are vital for mitochondrial integrity. Therefore, the process of mitochondrial translation, in which mtDNA‐encoded genes are translated into appropriate proteins, is crucial for mitochondrial function and biogenesis. Mitochondrial translation is divided into four phases: initiation, elongation, termination, and mitoribosome recycling (Christian and Spremulli 2012). In each phase, a series of mitochondrial translation factors, all of which are encoded by nDNA, is required for mtDNA‐encoded mitochondrial protein synthesis (Christian and Spremulli 2012). Mitochondrial translation factors coordinate each translational phase: the initiation phase uses mitochondrial initiation factor 2 and 3 (mtIF2 and mtIF3) (Ma and Spremulli 1995; Koc and Spremulli 2002; Overman et al. 2003; Christian and Spremulli 2009); the elongation phase requires the mitochondrial elongation factor Tu, Ts, and G1 (mtEFTu, mtEFTs, and mtEFG1) (Woriax et al. 1995; Xin et al. 1995; Gao et al. 2001; Hammarsund et al. 2001); the termination phase employs mitochondrial translational release factor 1‐like (mtRF1L) (Soleimanpour‐Lichaei et al. 2007); and the recycling phase uses mitochondrial recycling factor 1 and 2 (mtRRF1 and mtRRF2) (Zhang and Spremulli 1998; Tsuboi et al. 2009). Additionally, translational activators, such as translational activator of cytochrome c oxidase 1 (TACO1), bind mitochondrial mRNAs and regulate the expression levels of mitochondrial proteins (Weraarpachai et al. 2009; Richman et al. 2016). Clinically, mutations in *mtEFG1* (Coenen et al. 2004; Valente et al. 2007), *mtEFTs* (Smeitink et al. 2006), *mtEFTu* (Valente et al. 2007), and *TACO1* (Weraarpachai et al. 2009) are found in patients with severe mitochondrial translation deficit and dysfunction in oxidative phosphorylation; these patients are affected by clinical conditions such as lactic acidosis and fatal hypertrophic cardiomyopathy.

Exercise enhances mitochondrial biogenesis in skeletal muscle (Holloszy 1967; Holloszy and Booth 1976) through increases in the translation of both nDNA‐ and mtDNA‐encoded mitochondrial proteins. Therefore, mitochondrial translation factors are likely regulated by exercise. However, it is unclear whether exercise also affects mitochondrial translation factors in the skeletal muscle. A previous study reported that voluntary running under conditions of normal diet or high‐fat and high‐cholesterol diet altered the expression of mitochondrial translation factors in the murine gastrocnemius muscle (Lee et al. 2016). Exercise training significantly increased the levels of mtIF3, TACO1, and mtDNA‐encoded proteins, but not the levels of mtIF2 and mtEFTu, in mice fed a normal diet (Lee et al. 2016). Under the conditions of Western diet feeding, exercise training significantly increased the levels of mtIF3, mtEFTu, and TACO1, although those of mtIF2 were decreased (Lee et al. 2016). Another study reported that resistance training increases the levels of mtIF2, but decreases those of mtEFTu, in the rat gastrocnemius muscle (Greene et al. 2014), suggesting that exercise‐induced responses of mitochondrial translation factors does not appear to coincide with the expression of mitochondrial proteins. In contrast, integral mitochondrial biogenesis clearly requires the coordinated expression of both nDNA‐ and mtDNA‐encoded proteins. This indicates that exercise‐induced mitochondrial biogenesis likely coincides with the expression of mitochondrial translation factors.

In this study, we hypothesized that chronic exercise‐induced mitochondrial biogenesis coincides with the expression of mitochondrial translation factors. To test this hypothesis, we investigated whether exercise training comprehensively increases the levels of mitochondrial translation factors, as well as those of mtDNA‐encoded proteins, in the skeletal muscle.

## Methods

### Animals

All procedures and animal care were approved by the Committee on Animal Care at the Ritsumeikan University and were conducted in accordance with the Declaration of Helsinki. Male C57BL/6J mice were purchased from Shimizu Laboratory Supplies (Kyoto, Japan). Mice were singly housed under controlled conditions on a 12‐h light, 12‐h dark cycle and were provided food and water ad libitum.

To investigate the relationship between mtDNA‐encoded proteins and mitochondrial translation factors in metabolically heterogeneous hindlimb muscles (Benton et al. 2008), 10‐week‐old mice (*n* = 3) were initially acclimatized for 1 week and then sacrificed. Skeletal muscles (gastrocnemius, plantaris, soleus, extensor digitorum longus, and tibias anterior muscle) were rapidly excised, frozen in liquid nitrogen, and stored at −80°C until use.

### Exercise protocol

After one week of initial acclimatization, 8‐week‐old mice were randomly assigned to either a sedentary (*n* = 6) or an exercise group (*n* = 6). Each group of mice was housed in a standard cage with or without a running wheel for 1 or 8 weeks. Body weight was measured each week. The morning after the last day of the running period, the mice were deeply anesthetized with pentobarbital sodium; the muscle tissues were rapidly excised, frozen in liquid nitrogen, and stored at −80°C until use. To investigate the effects of exercise on the expression of mitochondrial translation factors in type I and type II‐rich muscles, synergist muscles (soleus and plantaris muscles) were analyzed as described previously (Ikeda et al. 2006; Wada et al. 2015). In pilot studies, we validated that our training protocol resulted in the progressive increase in running distance during exercise periods and increased endurance exercise capacity after chronic intervention.

### Antibodies

The following antibodies were used: mtIF2 (1:200, Santa Cruz Biotechnology, Dalla, TX, USA, sc‐365477); mtIF3 (1:2000, Origene, Rockville, MD, USA, TA800421); mtEFTu (1:5000, Abcam, Cambridge, UK, ab173300); mtEFTs (1:10,000, Abcam, ab173528); mtEFG1(1:3000, Abcam, ab173529); mtRF1L (1:1000, Proteintech, Rosemont, IL, 16694‐1‐AP); mtRRF1 (1:1000, Proteintech, 12357‐2‐AP); mtEFG2 (1:1000, Proteintech, 16941‐1‐AP); TACO1 (1:3000, Proteintech, 21147‐1‐AP); insulin receptor substrate 1 (IRS‐1, 1:3000, Cell Signaling Technology, Danvers, MA, USA, #3194); hormone sensitive lipase (HSL, 1:3000, Cell Signaling Technology, #18381); glyceraldehyde 3‐phosphate dehydrogenase (GAPDH, 1:10000, Wako, Osaka, Japan, 016‐25523); *β*‐tubulin (1:10,000, Wako, 014‐25041).

We used the Total OXPHOS Rodent WB Antibody Cocktail (1:3000, Abcam, ab110413) to detect the proteins involved in mitochondrial oxidative phosphorylation (OXPHOS). This cocktail contains five monoclonal antibodies: mitochondrial NADH dehydrogenase [ubiquinone] 1 beta subcomplex subunit 8 (NDUFB8, ab110242); mitochondrial succinate dehydrogenase [ubiquinone] iron‐sulfur subunit (SDHB, ab14714); mitochondrial cytochrome b‐c1 complex subunit 2 (UQCRC2, ab14745); mitochondrial encoded cytochrome c oxidase I (MTCO1, ab14705); mitochondrial ATP synthase subunit alpha (ATP5A, ab14748).

### Western blotting

Western blotting was performed as described previously (Yokokawa et al. 2015). The gastrocnemius (GA), plantaris (PLA), soleus (SOL), extensor digitorum longus (EDL), and tibias anterior (TA) muscles were lysed and homogenized in ice‐cold RIPA buffer (Cell Signaling Technology) supplemented with a protease and phosphatase inhibitor cocktail (Sigma‐Aldrich, St. Louis, MO, USA) and 0.1% sodium dodecyl sulfate. Next, the lysates were incubated for 60 min at 4°C with periodic vortexing. After centrifugation at 14,000*g* for 20 min at 4°C, the protein concentration of the supernatants was determined using a Protein Assay Bicinchoninate Kit (Nacalai Tesque, Kyoto, Japan). Lysates of equal volume (0.2–25 *μ*g) were subjected to SDS‐PAGE (7–13%), and then the separated proteins were transferred to 0.45‐*μ*m pore polyvinylidene difluoride membranes (Millipore, Bedford, MA, USA) at 100 V for 30 min. After blocking with 5% non‐fat dry milk (NFDM)/Tris‐buffered saline‐0.01% Tween 20 (TBST) for 30 min at 25°C, the membranes were washed twice (5 min each time) with TBST. Primary antibodies were diluted in 5% bovine serum albumin/TBST, 1% NFDM/TBST, or HIKARI solution A (Nacalai Tesque), and incubated overnight at 4°C. The membranes were then washed twice (5 min each time) with TBST and incubated with appropriate horseradish peroxidase‐linked secondary antibodies (1:1000–1:10,000, Cell Signaling Technology, 7074 or 7076) in 1–5% NFDM/TBST, TBST, or HIKARI solution B (Nacalai Tesque) for 60 min at 25°C. After washing with TBST 3–6 times (5 min each time), chemiluminescence quantification was performed using Laminata Forte Western HRP Substrate (Millipore), followed by image acquisition on an ImageQuant LAS 4000 (GE Healthcare, Little Chalfont, UK).

### Ponceau S staining

Following protein electrotransfer, the membranes were rinsed briefly with distilled water and incubated in Ponceau S staining solution (0.5% Ponceau S and 1% acetic acid in distilled water) for 10 min at 25°C. The stained membranes were washed briefly with distilled water, and digital images were acquired on an ImageQuant LAS 4000.

### Coomassie brilliant blue staining

The membranes were rinsed briefly with distilled water and stained in Coomassie Brilliant Blue (CBB) solution (0.25% CBB R‐250, 40% methanol, and 10% acetic acid) for 5 min at 25°C. The stained membranes were incubated in destaining solution (40% methanol and 10% acetic acid) three times (5 min each time) and then washed with distilled water. The membranes were then dried, and digital images were obtained on an ImageQuant LAS 4000.

### Image analysis and quantification

The images obtained via immunoblotting, Ponceau S staining, and CBB staining were analyzed using the Fiji image processing package based on ImageJ software (NIH, Bethesda, MD, USA) (Schindelin et al. 2012). The signal intensities of immunoblotting and CBB staining were independently calculated and converted to values relative to the gastrocnemius muscle and sedentary control.

### Statistical analysis

All statistical analyses were performed using open source R software (Ihaka and Gentleman 1996). Regardless of whether the compared groups exhibited homoscedasticity or heteroscedasticity, comparisons between the sedentary group and exercise group were performed using two‐tailed Welch's *t*‐tests. We used the Benjamini and Hochberg multiple comparisons method to compare protein expression levels among the five muscles. The Brown‐Forsythe test was used to evaluate homoscedasticity with the car package within R. The relationships between the expression of mtDNA‐encoded proteins and expression of mitochondrial translation factors were assessed using Pearson's product‐moment correlation coefficients. All barplots and scatterplots were produced using the ggplot package within R. The statistical significance level was set to *P *<* *0.05. All results are presented as dot plots and the means ± standard error of the mean (SEM).

## Results

### Relationship between endogenous mitochondrial translation factors and mitochondrial proteins in metabolically heterogeneous muscles

#### Mitochondrial proteins and mitochondrial translation factors

**Figure 1:**
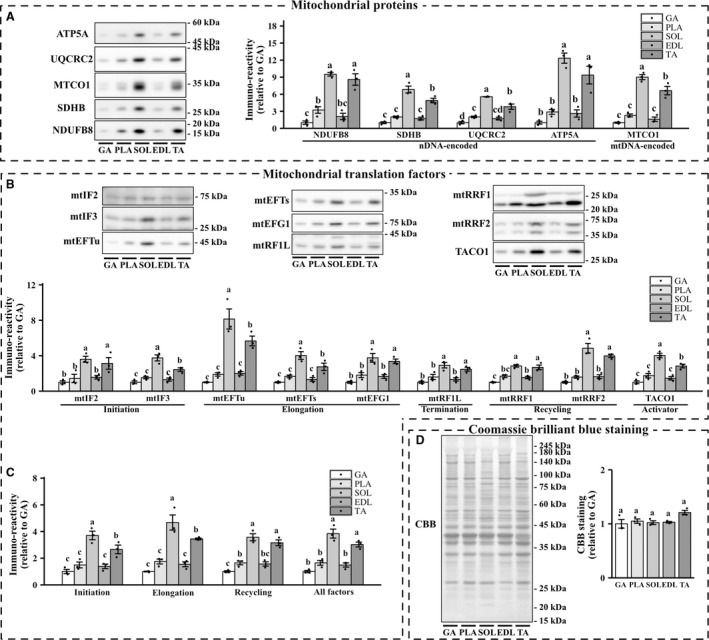
Protein expressions of mitochondrial translation factors in metabolically heterogeneous muscles. Representative image and quantification of mitochondrial proteins (A), mitochondrial translation factors (B and C), and CBB staining (D). Values are mean ± SEM; dot plot represents individual data points; *n *=* *3 per group.

**Figure 2:**
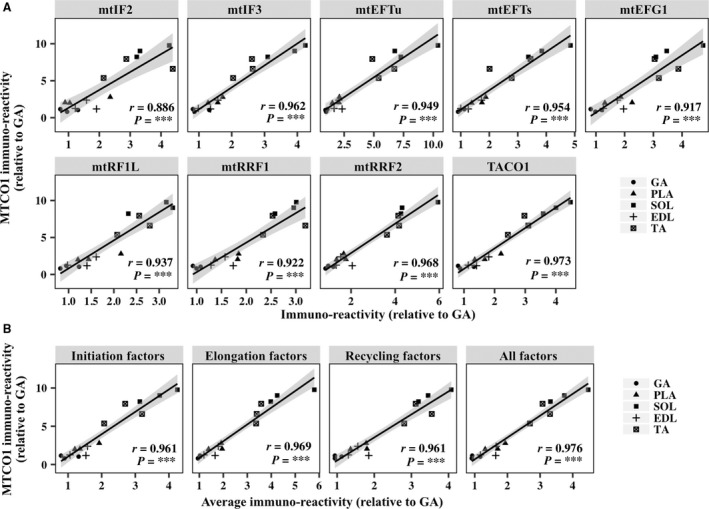
Correlation between the content of mtDNA‐encoded proteins and expression of mitochondrial translation factors in metabolically heterogeneous muscles. (A) Scatterplots show the levels of MTCO1 versus those of mitochondrial translation factors in the gastrocnemius (GA), plantaris (PLA), soleus (SOL), extensor digitorum longus (EDL), and tibias anterior (TA) muscles. (B) Scatterplots show the levels of MTCO1 versus the average expression of initiation factors, elongation factors, and recycling factors and total expression of all factors in GA, PLA, SOL, EDL, and TA muscles. The shaded areas show 95% confidence limits of the fitted line. *n *=* *3 per group. ***P <* *0.001.

Mitochondrial translation factors are crucial for mtDNA‐encoded protein expression and the integrity of mitochondrial functions. However, it remains unclear whether endogenous mitochondrial translation factors regulate the expression of mtDNA‐encoded proteins under physiological conditions. We first assessed the relationship between the expression levels of mitochondrial translation factors and protein content of MTCO1, a mtDNA‐encoded protein, in the five skeletal muscles. The expression of mitochondrial OXPHOS proteins (Fig. 1A) and mitochondrial translation factors (Fig. 1B) in red oxidative muscles, particularly in the soleus muscle, was higher than that in white glycolytic muscles. Similarly, the average expression levels of initiation factors (mtIF2 and mtIF3), elongation factors (mtEFTu, mtEFTs, and mtEFG1), recycling factors (mtRRF1 and mtRRF2), and the total expression of all factors in the red oxidative muscles were higher than those in white glycolytic muscles (Fig. 1C). We found significant correlations between the expression levels of all mitochondrial translation factors and MTCO1 protein (Fig. 2A). Correspondingly, the average expression levels of initiation factors, elongation factors, and recycling factors and total expression of all factors were significantly correlated with MTCO1 protein levels (Fig. 2B).

#### CBB staining

We conducted Coomassie Brilliant Blue (CBB) staining to confirm equal protein loading. No statistically significant difference in CBB staining was detected among the five skeletal muscles (Fig. 1D), and similar results were reproduced using Ponceau S staining (data not shown).

### Effect of 8‐week exercise training on expression of mitochondrial translation factors

#### Physiological characteristics

**Figure 3:**
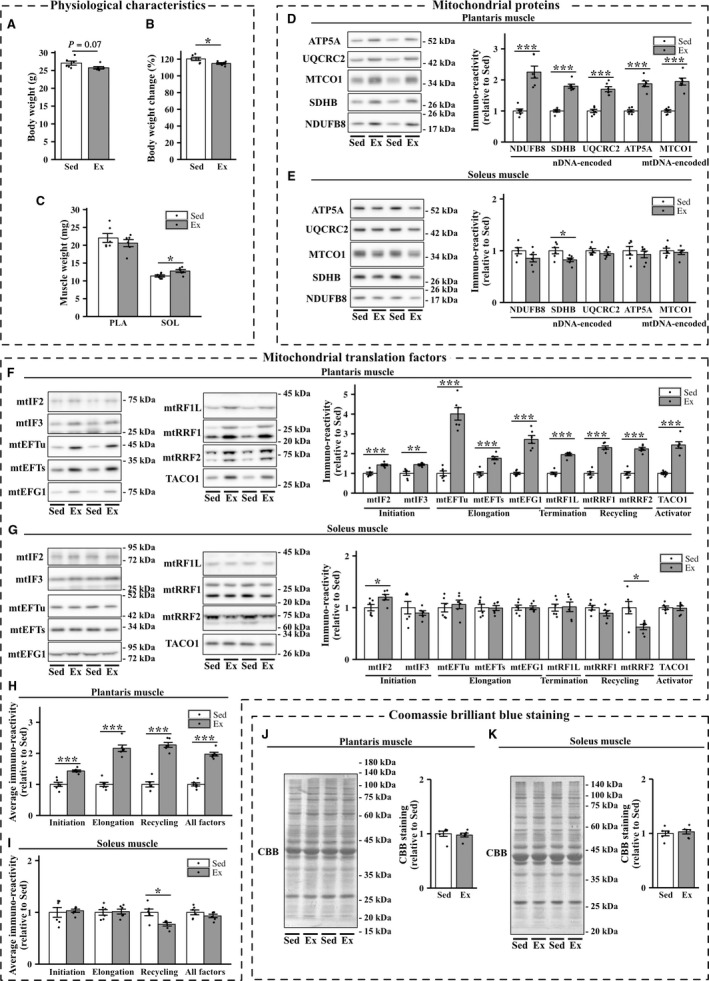
Effects of 8‐week exercise training on the expression of mitochondrial proteins and mitochondrial translation factors in the plantaris and soleus muscles. Body weight (A), body weight change (B), and muscle weight (C) in sedentary mice (Sed) and exercised mice (Ex). (D–G) Representative immunoblots and quantifications of OXPHOS protein levels (D and E) and those of mitochondrial translation factors (F and G) in the plantaris and soleus muscles of sedentary and exercised groups of mice. (J and K) Representative images and quantifications of Coomassie brilliant blue (CBB) staining. Values are mean ± SEM; dot plot represents individual data points; *n *=* *6 per group. *P <* *0.05 vs. Sed. **P <* *0.01 vs. Sed. ***P <* *0.001 vs. Sed.

The body weight change in the exercise group was significantly lower than that in the sedentary group (Fig. 3B). No significant differences in the weight of the plantaris muscle were found between the two groups. In contrast, the weight of soleus muscle in exercised mice was significantly higher than that in sedentary mice (Fig. 3C).

#### Mitochondrial proteins and mitochondrial translation factors

**Figure 4:**
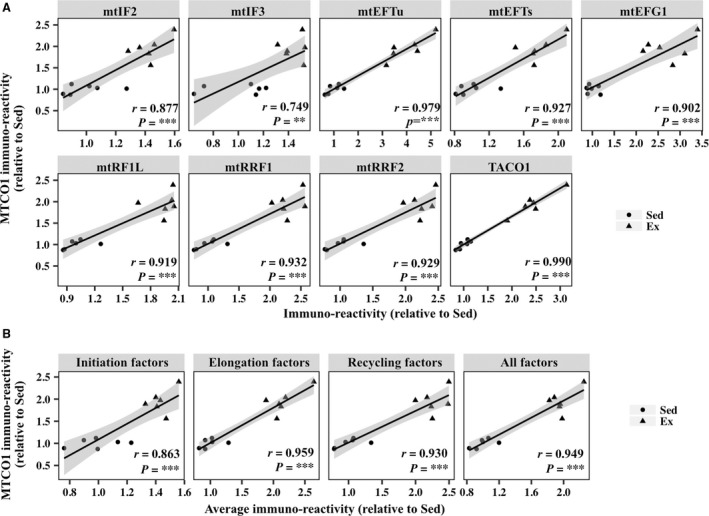
Correlation between the levels of mtDNA‐encoded protein MTCO1 and mitochondrial translation factors in the plantaris muscle of sedentary mice (Sed) and mice exercised for 8 weeks (Ex). (A) Scatterplots showing the levels of MTCO1 versus those of mitochondrial translation factors. (B) Scatterplots showing the levels of MTCO1 versus average levels of initiation factors, elongation factors, and recycling factors and total levels of all the factors. The shaded areas show 95% confidence limits of the fitted line. *n *=* *6 per group. **P <* *0.01. ***P <* *0.001.

Exercise training for 8 weeks significantly increased the levels of a mtDNA‐encoded protein (MTCO1), as well as NDUFB8, SDHB, UQCRC2, and ATP5A, all of which are nDNA‐encoded mitochondrial proteins, in the plantaris muscle (Fig. 3D). We did not detect exercise‐induced increases in nDNA‐ or mtDNA‐encoded mitochondrial proteins in the soleus muscle, which showed a slight, but significant, decrease in the protein content of SDHB in the exercise group (Fig. 3E). Eight‐week exercise training elevated the expression levels of initiation factors (mtIF2 and mtIF3), elongation factors (mtEFTs, mtEFTu, and mtEFG1), the release factor mtRF1L, recycling factors (mtRRF1 and mtRRF2), and translational activator TACO1 in the plantaris muscle (Fig. 3F). Mitochondrial translation factors exhibited significant positive correlations with the levels of MTCO1 in the plantaris muscle (Fig. 4A). Furthermore, the average expression of initiation factors, elongation factors, and recycling factors and total expression of all the factors were clearly correlated with the levels of MTCO1 (Fig. 4B). In the soleus muscle, exercise training for 8 weeks increased the protein expression of mtIF2. However, 8‐week exercise training did not significantly change the protein expression of mtIF3, mtEFTs, mtEFTu, mtEFG1, mtRF1L, mtRRF1, and TACO1 (Fig. 3, G and I). Unexpectedly, the protein expression of mtRRF2 was downregulated in the soleus muscle of exercised mice.

#### CBB staining

Exercise training significantly increased the expression of *β*‐tubulin and decreased that of GAPDH protein in the soleus muscle (data not shown). Therefore, we performed CBB and Ponceau S staining to verify equal protein loading. In the plantaris and soleus muscles, no statistically significant difference in CBB staining was found between the two groups (Fig. 3, J and K). Similar results were obtained by Ponceau S staining (data not shown).

### Effect of 1‐week exercise training on the expression of mitochondrial translation factors

#### Physiological characteristics

**Figure 5:**
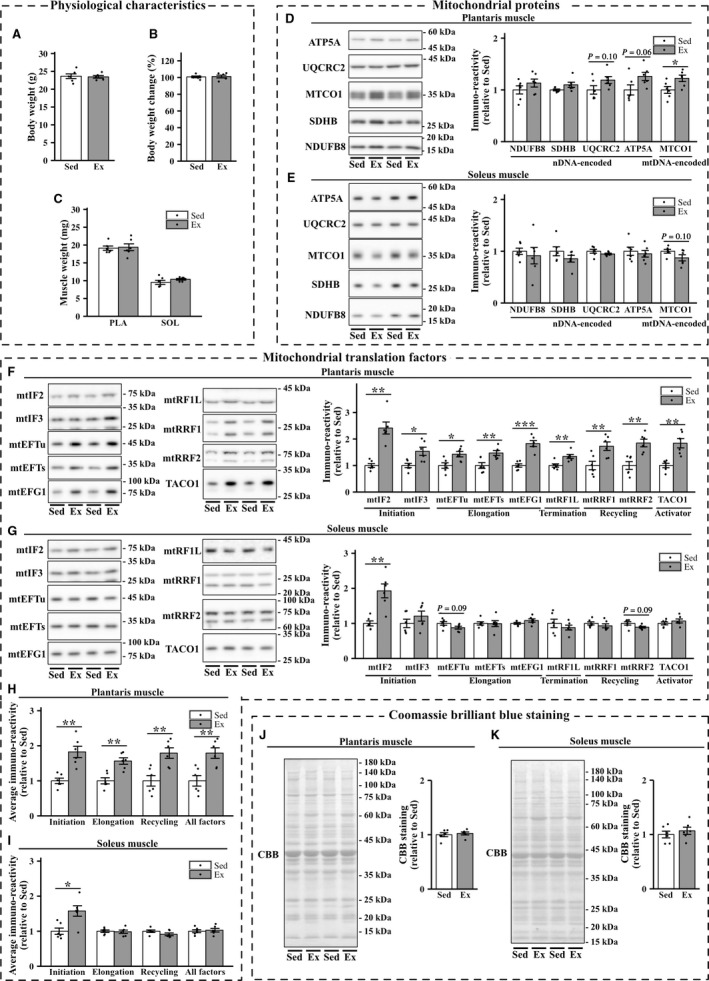
Effects of 1‐week exercise training on the expression of mitochondrial proteins and mitochondrial translation factors in the plantaris and soleus muscles. Body weight (A), body weight change (B), and muscle weight (C) in sedentary mice (Sed) and exercised mice (Ex). (D–G) Representative immunoblots and quantifications of OXPHOS protein levels (D and E) and those of mitochondrial translation factors (F and G) in the plantaris and soleus muscles of sedentary and exercised groups of mice. (J and K) Representative images and quantifications of Coomassie brilliant blue (CBB) staining. Values are mean ± SEM; dot plot represents individual data points; *n *=* *6 per group. *P <* *0.05 vs. Sed. **P <* *0.01 vs. Sed. ***P <* *0.001 vs. Sed.

No significant differences in body weight, body weight change, and muscle weight were detected between the sedentary group and group exercised for 1 week (Fig. 5A–C).

#### Mitochondrial proteins and mitochondrial translation factors

**Figure 6:**
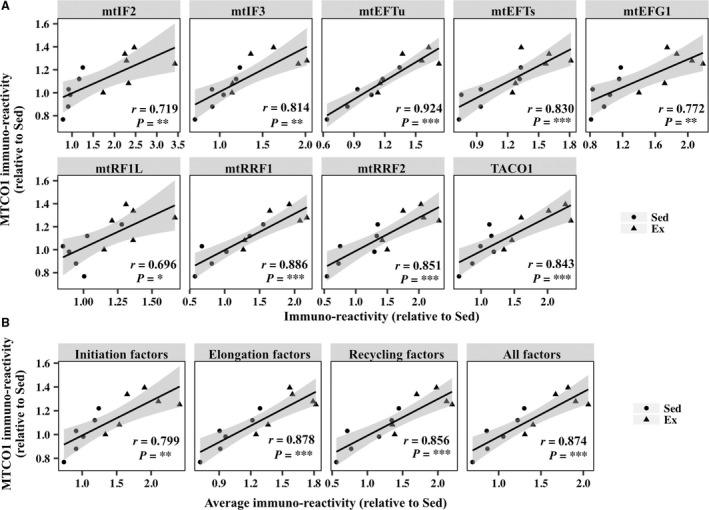
Correlation between the levels of mtDNA‐encoded protein MTCO1 and those of mitochondrial translation factors in the plantaris muscles of sedentary mice (Sed) and mice exercised for 1 week (Ex). (A) Scatterplots of the levels of MTCO1 versus those of mitochondrial translation factors. (B) Scatterplots of the levels of MTCO1 versus average levels of initiation factors, elongation factors, and recycling factors and total levels of all the factors. The shaded areas show 95% confidence limits of the fitted line. *n* = 6 per group. *P < 0.05 vs. Sed. **P < 0.01. ***P < 0.001.

Exercise training for 1 week significantly increased the content of MTCO1, and showed a tendency to increase NDUFB8, SDHB, UQCRC2, and ATP5A, in the plantaris muscle (Fig. 5D). In the soleus muscle, exercise‐induced increases in the levels of nDNA‐ or mtDNA‐encoded mitochondrial proteins were not detected (Fig. 5E). One‐week exercise training elevated the expression levels of all mitochondrial translation factors in the plantaris muscle (Fig. 5F). All mitochondrial translation factors showed significant positive correlations with MTCO1 protein content in the plantaris muscle (Fig. 6A and B). In the soleus muscle, exercise training for 1 week increased the expression of mtIF2. However, other factors were not significantly modified and a decreasing trend was observed for mtRRF2 (Fig. 5G).

#### CBB staining

In the plantaris and soleus muscles, no statistically significant differences in CBB staining were observed between the two groups (Fig. 5J and K). Similar results were obtained by Ponceau S staining (data not shown).

## Discussion

Our results indicate that endogenous expression levels of mtDNA‐encoded proteins are closely related to the expression of mitochondrial translation factors in the skeletal muscle. Our results also show that exercise‐induced mitochondrial biogenesis in the muscle occurs concomitantly with comprehensive expression of mitochondrial translation factors. In this study, exercise training enhanced the expression of nDNA‐ and mtDNA‐encoded mitochondrial proteins and mitochondrial translation factors in the plantaris muscle. In the soleus muscle, exercise‐induced increases in the levels of mitochondrial proteins and translation factors were not detected; however, a slight but significant decrease in the protein content of SDHB and mtRRF2 was observed in the exercise group. This is the first study to show that exercise training comprehensively regulates the expression of mitochondrial translation factors in the plantaris muscle.

Our results show that exercise training increased nDNA‐ and mtDNA‐encoded mitochondrial protein levels in the plantaris muscle. In the soleus muscle, exercise training did not increase mitochondrial proteins as muscle weight increased, which is consistent with the results of a previous study (Ikeda et al. 2006). Several studies have shown that exercise on a running wheel does not induce mitochondrial adaptation in the soleus muscle (Hokari et al. 2010) (Rowe et al. 2012). Although the physiological and molecular mechanisms underlying this phenomenon are unclear, the lack of mitochondrial adaptation in the soleus muscle may stem from the difference in muscular recruitment under the conditions of running on a wheel. Additionally, muscle hypertrophy increases the content of hypertrophy‐related proteins, such as cytoskeletal proteins, and may mask increases in the absolute amount levels of mitochondrial proteins in the soleus muscle. Indeed, we found that exercise training decreased the levels of glycolytic enzyme GAPDH and increased those of the cytoskeletal protein *β*‐tubulin. Intramuscularly, the relative content of mitochondrial proteins did not increase in the soleus muscle of the exercised group; however, the wheel running model may be insufficient for investigating the absolute content of mitochondrial proteins in the soleus muscle. Thus, further studies using other exercise models are necessary to determine the fiber type‐specific responses of the absolute levels of mitochondrial proteins under conditions of exercise training.

Exercise training also upregulated mitochondrial translation factors in the plantaris muscle, which was concomitant with increases in the levels of nDNA‐ and mtDNA‐encoded mitochondrial proteins. In the soleus muscle, these increases were not detected; however, slight downregulation of mtRRF2 levels was detected in exercised mice. Although there were several minor differences, these results were reproduced in challenges using 1‐ and 8‐week exercise regiments. A single‐week exercise study was performed to avoid chronic exercise‐induced indirect factors (e.g. body weight and muscle weight change) and detect increases in mitochondrial translation factors in the absence of body weight change, unlike 8‐week exercise. Thus, indirect factors were unlikely to alter the content of mitochondrial translation factors. These results suggest that these proteins accumulate during prolonged exercise periods.

Our results indicate that exercise training comprehensively increases the relative content of mitochondrial translation factors, concomitant with that of mitochondrial proteins, in the plantaris muscle but not in the soleus muscle. Exercise‐induced adaptation of the expression of mitochondrial translation factors may coincide with the expression of mitochondrial proteins. In contrast, previous studies showed that exercise decreases the protein expression of mtEFTu in the rat gastrocnemius muscle (Greene et al. 2014), and that 4‐week running exercise training does not significantly increase the protein content of mtIF2 and mtEFTu in the murine gastrocnemius muscle (Lee et al. 2016); these results are partly inconsistent with those of the current study and failed to detect comprehensive increases in mitochondrial translation factors with mtDNA‐encoded proteins. These inconsistencies may be related to differences in the exercise models and training periods. Additionally, in previous studies, muscle samples were homogenized in lysis buffer that did not contain detergent, unlike our protocol, and were then centrifugated at 10,000*g* for 30 min. Therefore, large numbers of mitochondria may have been lost from the supernatant, which was utilized for western blotting, leading to crucial technical errors. Several clinical studies showed that genetic alterations in *mtEFG1* (Coenen et al. 2004; Valente et al. 2007), *mtEFTs* (Smeitink et al. 2006), *mtEFTu* (Valente et al. 2007), and *TACO1* (Weraarpachai et al. 2009) cause severe mitochondrial translation deficit and OXPHOS dysfunction, suggesting that mitochondrial translation factors are crucial to the integrity of mitochondrial function. Therefore, these comprehensive adaptations may also be crucial factors in exercise‐induced mitochondrial biogenesis in the skeletal muscles. In the soleus muscle, exercise training specifically increased mtIF2 protein levels and decreased mtRRF2 protein levels. Therefore, the regulation of mtIF2 and mtRRF2 may differ from that of other translation factors. Further studies are needed to clarify the molecular mechanism by which exercise regulates the expression of mitochondrial translation factors.

A previous study reported that denervation‐induced muscle inactivity decreases mitochondrial biogenesis‐related proteins at 3 days after operation (Tryon et al. 2015). Additionally, several nDNA‐encoded mitochondrial proteins in rodent skeletal muscle are increased for a few hours after exercise (Wright et al. 2007). These previous studies suggest that mitochondria proteins rapidly adapt to physiological stimuli; therefore, we sacrificed the mice immediately after exercise to limit the effect of exercise cessation, which is thought to rapidly recover the increased mitochondrial proteins. Our result does not rule out the possibility that increases in the targets were derived from the residual effects of the last bout of exercise rather than from chronic effects. Although 8‐week exercise clearly increased mitochondrial translation factor levels in the plantaris muscle compared to 1‐week exercise, suggesting that the accumulation of chronic exercise effects regulates the content of mitochondrial translation factors, the present study did not clearly distinguish between chronic effects and acute effects. Therefore, further studies are required to investigate whether a single bout exercise affects mitochondrial translation factors.

In conclusion, our present results demonstrate that exercise training induced comprehensive upregulation of mitochondrial translation factors, which was concomitant with increased levels of mitochondrial proteins, in the plantaris muscle. These comprehensive adaptations were not detected in the soleus muscle. Thus, exercise‐induced mitochondrial biogenesis coincides with the expression of mitochondrial translation factors, which may be crucial for the integrity of mitochondrial function.

## Conflict of Interest

No conflicts of interest, financial or otherwise, are declared by the authors.
